# Supplementary material for: Identification of polarized macrophage subsets in zebrafish
Source: eLife. 2015 Jul 8;4:e07288. doi: 10.7554/eLife.07288 (PMC4521581; doi:10.7554/eLife.07288)
Supplement: Supplementary file 1. — Genes, accession numbers, and sequences of the primers. DOI: http://dx.doi.org/10.7554/eLife.07288.014 [file elife07288s001.pdf]

Supplementary file 1: Genes, accession numbers and sequences of the primers.

zebrafish primer				
Gene	Accession	Name primer	Nucleotide sequence (5'→3')	Use
tnfa promoter	Gene ID: 405785	zTNFaP4	CCCGCATGCTCCACGTCTCC	cloning
		zTNFaE11N	TTATAGCGGCCGCCCGACTCTCAAG CTTCA	
ccr2	ENSDARG 000000798 29	CCR2.5	TGGCAACGCAAAGGCTTTCAGTGA	qPCR
		CCR2.3	TCAGCTAGGGCTAGGTTGAAGAG	
il6	NM_00126 1449.1	IL6.5	TGAAGACACTCAGAGACGAGCAGTT	qPCR
		IL6.3	AGGTTTGAGGAGAGGAGTGCTGAT	
il10	NM_00102 0785	IL10.52	TCAGAGCAGGAGAGTCGAATGCA	qPCR
		IL10.32	CGATTGGGGTTGTGGAGTGCTT	
tgfb1	ENSDARG 000000415 02	TGFb1a 5.3	CAACCGCTGGCTCTCATTTGA	qPCR
		TGFb1a 3.3	ACAGTCGCAGTATAACCTCAGCT	
tnfb	NM_00102 4447.1	zTNFalp.51	CGAAGAAGGTCAGAAACCCA	qPCR
		zTNFalp.3	GTTGGAATGCCTGATCCACA	
tnfa	NM_21285 9	zTNFa.54	TTCACGCTCCATAAGACCCA	qPCR
		zTNFa.34	CCGTAGGATTCAGAAAAGCG	
		TNFa .55	GGCGTTTTTGGATGTTGAAG	probe
		TNFa. 38	TGCCCAGTCTGTCTCCTTCT	
mpeg1	NM_21273 7.1	zmpeg1.5	GTGAAAGAGGGTTCTGTTACA	qPCR
		zmpeg1.3	GCCGTAATCAAGTACGAGTT	
il1b	NM_21284 4.2	zIL1b.5	TGGACTTCGCAGCACAAAATG	qPCR
		zIL1b.3	GTTCACTTCACGCTCTTGGATG	
ef1a	ENSDARG 000000208 50	zEF1a.5	TTCTGTTACCTGGCAAAGGG	qPCR
		zEF1a.3	TTCAGTTTGTCCAACACCCA	
arg1	NM_00104 5197.1	ARG1 5.3	ACGGCCAGCCGATGTCTTAC	qPCR
		ARG1 3.3	TCCACGTCTCGGAGTCCAAT	
